# The Difluoroboranyl-Fluoroquinolone Derivative “7a” Inhibits Bacterial DNA Gyrase and Exhibits Potent Activity Against Ciprofloxacin-Resistant *S. aureus* In Vitro and In Vivo Using an Acute Pneumonia Model

**DOI:** 10.3390/molecules31061044

**Published:** 2026-03-20

**Authors:** Luis Angel Veyna-Hurtado, Hiram Hernández-López, Denisse de Loera, Juan Manuel Vargas-Morales, Martín Muñoz-Ortega, Lorena Troncoso-Vázquez, Alondra Bocanegra-Zapata, Alberto Rafael Cervantes-Villagrana

**Affiliations:** 1 Facultad de Ciencias Químicas, Universidad Autónoma de San Luis Potosí, San Luis Potosí 78210, Mexico; angelveyna@gmail.com (L.A.V.-H.); abocanegrazapata@gmail.com (A.B.-Z.); 2 Laboratorio de Investigación en Síntesis Orgánica y Modificación Química, Unidad Académica de Ciencias Químicas, Universidad Autónoma de Zacatecas, Zacatecas 98160, Mexico; hiram.hernandez.lopez@uaz.edu.mx; 3 Laboratorio de Análisis Clínicos, Facultad de Ciencias Químicas, Universidad Autónoma de San Luis Potosí, San Luis Potosí 78100, Mexico; juan.vargas@uaslp.mx; 4 Laboratorio de Patología Molecular, Departamento de Química, Universidad Autónoma de Aguascalientes, Aguascalientes 20100, Mexico; humberto.munozo@edu.uaa.mx; 5 Departamento de Patología, Instituto Mexicano del Seguro Social (IMSS), Zacatecas 99000, Mexico; lorecrm@hotmail.com; 6 Laboratorio de Investigación en Terapéutica Experimental, Unidad Académica de Ciencias Químicas, Universidad Autónoma de Zacatecas, Zacatecas 98160, Mexico

**Keywords:** fluoroquinolone, *S. aureus*, resistance, molecular docking, pneumonia, toxicity, histopathology

## Abstract

According to the World Health Organization, antibiotic research remains insufficient, emphasizing the urgent need for new active molecules, particularly against resistant bacteria. Based on known antibacterial scaffolds, new fluoroquinolone derivatives have been synthesized by our research group, including compound **7a**, a difluoroboranyl-fluoroquinolone that previously demonstrated activity against sensitive strains. Methods: The minimum inhibitory (MIC) and bactericidal (MBC) concentrations of compound **7a** were determined against *Staphylococcus aureus*, *Klebsiella pneumoniae*, and *Escherichia coli*. The selective development of ciprofloxacin-resistant *S. aureus* was induced by reseeding the isolate on seven consecutive days with an antibiotic concentration that was not capable of inhibiting its development. Pharmacokinetic and toxicological properties were predicted using SwissADME, Way2Drug, and molecular docking (AutoDock Vina). In vivo toxicity was evaluated in BALB/c mice through histopathological liver and kidney analysis and serum biochemical markers. The antibacterial efficacy of **7a** (80 mg/kg/day) was assessed in a murine pneumonia model induced by ciprofloxacin-resistant *S. aureus*. DNA gyrase inhibition was confirmed through plasmid electrophoresis assays in *E. coli* DH5-α cells. Results: Compound **7a** exhibited both MIC and MBC values of 0.25 μg/mL, while ciprofloxacin-resistant *S. aureus* strains did not exhibit a detectable MIC within the concentration range tested (up to 1024 μg/mL). In silico predictions revealed favorable ADME profiles, low toxicity, and strong interaction with DNA gyrase. *In vivo*, **7a** showed no signs of hepatotoxicity or nephrotoxicity and effectively reduced pneumonic tissue to 1.99% in infected mice. Electrophoretic assays confirmed DNA gyrase inhibition consistent with the mechanism of fluoroquinolones. Conclusions: Compound **7a** evidenced activity against ciprofloxacin-resistant *S. aureus* in vitro and reduced infection progression in vivo. It also displays favorable drug-like properties, low predicted toxicity, and DNA gyrase inhibition.

## 1. Introduction

Millions of microorganisms constantly coexist with humans, with bacteria being the most widespread group and responsible for a large number of infectious diseases associated with high morbidity and mortality [1]. The introduction and application of antibiotics provided an effective tool for significantly reducing mortality from infectious diseases [2,3].

However, bacteria have developed various resistance mechanisms that allow them to reduce the intracellular accumulation of antibiotics, such as the overexpression of efflux pumps or modification of the molecular target [4,5]. This phenomenon has been greatly favored by the empirical administration and abuse of these therapeutic agents, decreasing the efficacy of antibiotics [6].

In response to this scenario, the World Health Organization (WHO) has recognized this threat to public health and has called for intensified research and development of new antibacterial drugs [7]. The low number of compounds reaching clinical phases contrasts with the rapid increase in resistant strains, creating a critical situation that has been described as an imminent “post-antibiotic era” [3,7,8,9].

Recently, it was reported by the WHO that antimicrobial resistance (AMR) was associated with approximately 4.95 million deaths in 2019, with 1.27 million deaths directly attributable to resistant bacteria, where the microorganisms with the greatest impact were *E. coli*, *Staphylococcus aureus*, *Klebsiella pneumoniae*, *Streptococcus pneumoniae*, *Acinetobacter baumannii* and *Pseudomonas aeruginosa*; projections estimate that without urgent action, AMR could cause 39 million deaths between 2025 and 2050 [10,11]. In addition, methicillin-resistant *S. aureus* (MRSA) has been shown to be a pathogen that is widely spread in hospitals and the community, whose drug resistance makes it a risk factor in skin and soft tissue infections, pneumonia, and sepsis [12]. The emergence and dissemination of ciprofloxacin-resistant *S. aureus* underscore the urgent need for alternative antimicrobial strategies and the development of novel compounds capable of overcoming fluoroquinolone resistance.

In this context, quinolone-derived compounds are a promising avenue in current research, allowing them to be used as a basis for obtaining new compounds with significant bactericidal activity, especially against the bacteria mentioned above [13,14]. For this reason, our research group has synthesized a series of fluoroquinolones with the particularity of having a complex with boron atoms in positions 3 and 4 through keto and carboxylic groups [15]. In silico studies have reported that norfloxacin-boron complexes can increase affinity for DNA gyrase, the pharmacological target of this family of antibiotics, as well as improve their pharmacokinetic properties [16,17,18]. Therefore, this investigation focused on assessing a boron-complex-bearing compound to explore whether the incorporation of this residue may confer improved properties compared with conventional quinolone structures, particularly when tested against resistant bacterial strains such as *Staphylococcus aureus*.

**Figure 1:**
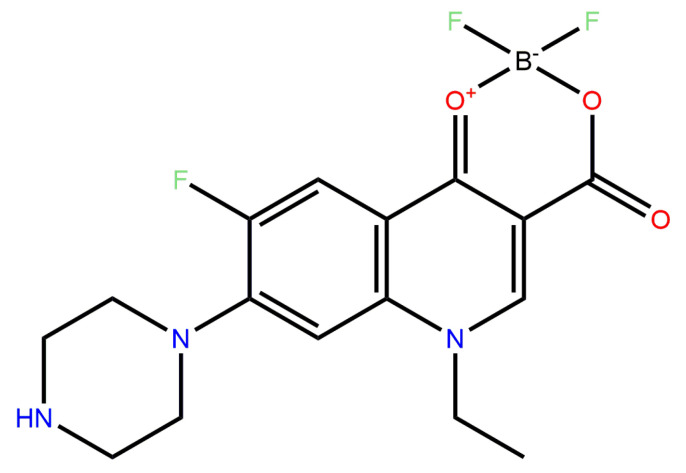
Chemical Structure of **7a** compound. The difluorinated boron complex was added at positions 3,4 of the fluoroquinolone, modifying the main interaction site of the drug family.

Such compounds have been extensively evaluated, and through in vitro screening, the compound difluoroboranyl 1-ethyl-6-fluoro-4-oxo-7-piperazin-1-yl-1,4-dihydro-quinoline-3-carboxylate, later labeled “**7a**” (shown in Figure 1), having demonstrated antibacterial potency and effectiveness against susceptible strains [17,19], However, until now, the antibacterial potential of this fluoroquinolone against strains resistant to ciprofloxacin had not been reported.

The development of new molecules with pharmacological potential has benefited from strategies that allow for a more efficient search for candidate compounds. Among these in silico simulations are a fundamental tool for estimating pharmacodynamic properties, metabolic pathways, and possible toxicity profiles [20,21,22]. This type of analysis helps optimize the use of biological and experimental resources, directing efforts toward the most promising compounds. However, the results obtained in silico must subsequently be validated through in vitro and in vivo models, which can confirm their effectiveness and safety in real biological systems, allowing reproducible information to be obtained on new molecules with potential pharmacological use.

Therefore, the main objective of this study was to evaluate the safety and efficacy of compound **7a** against ciprofloxacin-resistant *Staphylococcus aureus* (clinical isolate HGZ2201#ID) using a comprehensive experimental approach, integrating in vitro, in silico, and in vivo evaluations. For the latter, a murine model of acute pneumonia was used to examine the therapeutic performance of the compound under physiological conditions. In parallel, a molecular analysis of the inhibition of bacterial DNA gyrase by compound **7a** was performed to corroborate a possible mechanism of action consistent with that described for quinolones.

## 2. Results

In this study, the antimicrobial potential of compound ***7a*** was assessed, revealing a marked inhibitory effect against the tested bacterial strains.

### 2.1. Compound **7a** Interacts In Silico with S. aureus DNA Gyrase

**Figure 2:**
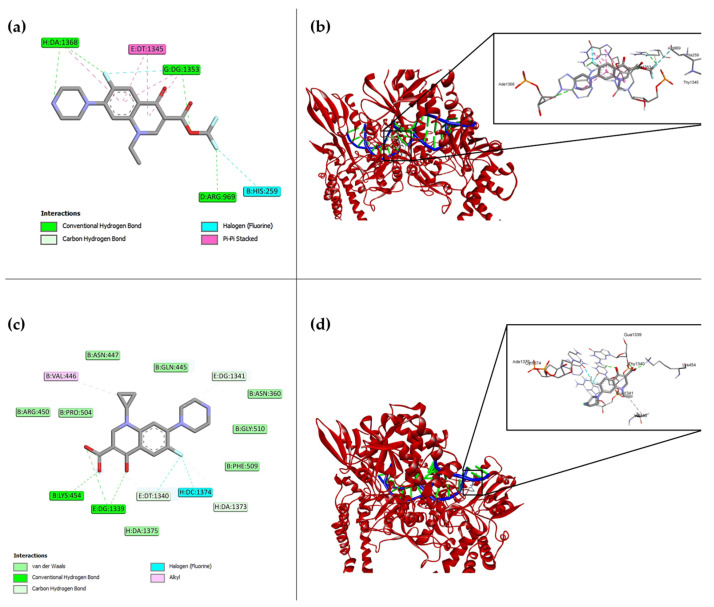
Molecular docking analysis of compound **7a** and ciprofloxacin (Cpx) with *S. aureus* DNA gyrase (PDB ID: 2xct). (**a**) Predicted binding interactions of compound **7a** with gyrase amino acid residues, showing a docking score of −10.0 kcal/mol. (**b**) Three-dimensional view of the **7a**–gyrase complex highlighting the optimal binding pose (black rectangle). (**c**) Predicted interactions between ciprofloxacin and DNA gyrase with a docking score of −8.9 kcal/mol. (**d**) Structural representation of the ciprofloxacin–gyrase complex indicating the best docking orientation.

In silico analysis of compound **7a** on the GyrA subunit of bacterial DNA gyrase (PDB ID: 2XCT) allowed us to identify the most probable binding conformations at the receptor’s catalytic site. The docking study generated nine possible orientations with their respective binding energy values, presented in Table 1. The most stable complex corresponded to position 1 of compound **7a**, with a score of −10.0 kcal/mol, higher than that observed for ciprofloxacin, suggesting a more favorable interaction affinity, as we can see in Figure 2a. Furthermore, the best interaction found is represented at Figure 2b, in a three-dimensional conformation with the gyrase.

Key residues involved in the interaction of **7a** with GyrA included **Arg969**, **His259**, **Gua1353**, **Thy1345**, **Phe970**, and **Ade1368**, stabilized by hydrogen bonds and van der Waals forces, as well as additional electrostatic and hydrophobic interactions (Figure 2a). Interestingly, the boron-containing moiety of **7a** established unique contacts with **His259** and **Arg969**, indicating a distinct binding orientation compared to classical fluoroquinolones.

In contrast, ciprofloxacin got the score −8.9 with their best conformation and formed hydrogen bond and van der Waals interactions mainly with **Lys454**, **Thy1340**, **Gua1339,** and **Pro504** (Figure 2c), and their three-dimensional interaction with the gyrase is represented at Figure 2d. Although both ligands shared mechanistic similarities, the interaction pattern of compound **7a** revealed an alternative binding geometry that may enhance its inhibitory potential toward DNA gyrase, consistent with the expected fluoroquinolone mechanism of action. Further information regarding the hydrophobic surface of the compound at the interaction site can be found in Supplementary Material, Figure S1.

### 2.2. Compound ***7a*** Shows Potency Equivalent to Ciprofloxacin Based on MIC and MBC

Regarding the determination of the Minimum Inhibitory Concentration (MIC), ciprofloxacin—used as the reference control—showed an MIC of 0.25 μg/mL and an MBC of 0.5 μg/mL against *S. aureus*. Similarly, the synthesized derivative **7a** exhibited an MIC of 0.25 μg/mL, with an equivalent MBC value of 0.25 μg/mL (Table 2). These results indicate that compound **7a** displays potent antibacterial activity against *S. aureus*, consistent with the CLSI criteria for active agents (MIC ≤ 1 μg/mL) [23].

The inhibitory capacity of the compounds was also determined with respect to the inhibition of strains obtained from clinical isolates of K. pneumoniae, where an identical MIC was again obtained for both **7a** and Cpx, but with a lower MBC in the case of **7a** (1 μg/mL). Such determinations were also obtained for a clinical isolate of *E. coli*, requiring 4 µg/mL for inhibition by **7a**, but with both **7a** and Cpx obtaining the same MBC of 8 µg/mL, as can be seen in Table 2.

**Figure 3:**
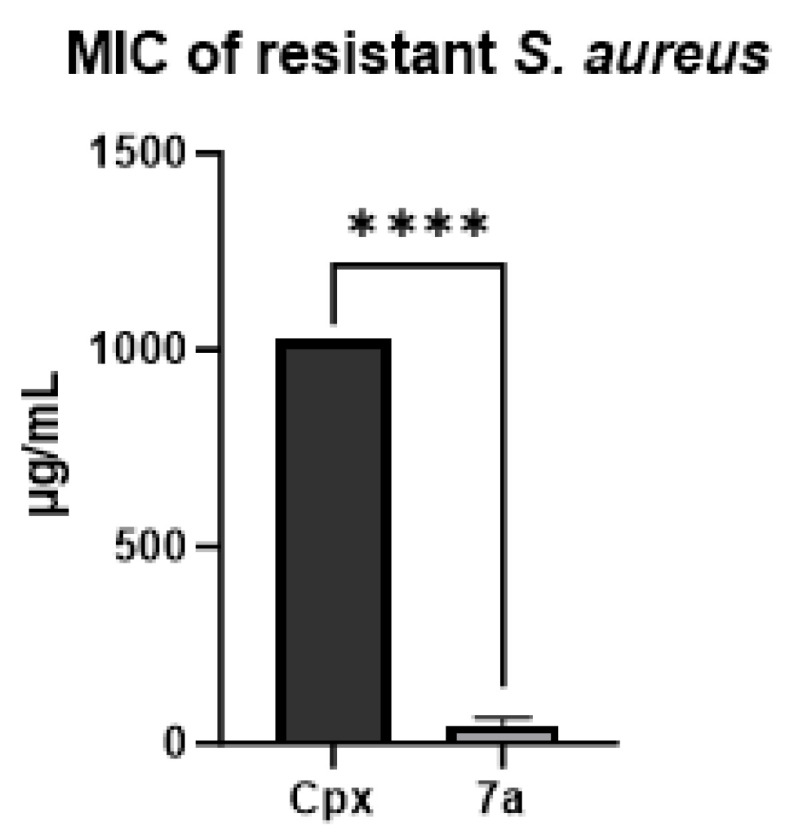
Final MIC against *S. aureus* resistant to ciprofloxacin. Mean and standard deviation of MIC found for ciprofloxacin and **7a**, performed in triplicate. Statistical analysis *T*-test, **** *p* < 0.0001.

The results demonstrated that compound **7a** exhibited antibacterial potency comparable to that of ciprofloxacin, used as the fluoroquinolone positive control. Based on these findings, a resistance-induction assay was performed using *S. aureus* cultures subjected to serial passages in the presence of a sub-inhibitory concentration (0.125 μg/mL) of the drug. After 14 consecutive passages, the MIC values were reassessed. Ciprofloxacin showed a marked increase in resistance, with a final MIC of 1024 μg/mL, whereas compound **7a** retained inhibitory activity against the resistant *S. aureus* strain at 32 μg/mL, as illustrated in Figure 3.

The results regarding the activity of compound **7a** on the strain with resistance to ciprofloxacin shed light on the potential of our experimental molecule as an antibacterial agent. We therefore conducted a series of in silico evaluations to predict its pharmacokinetic properties.

### 2.3. Compound ***7a*** Possesses Suitable In Silico ADME Properties Consistent with Future Drug Potential

The SwissADME web server allows for rapid calculation of key ADME parameters, such as lipophilicity (iLOGP), solubility (log S), topological polar surface area (TPSA), gastrointestinal absorption, P-glycoprotein substrate status, and overall bioavailability radar, pharmacokinetic properties relevant to a potential drug and its oral administration. Some of these main parameters can be seen in Table 3.

The bioavailability obtained for both **7a** and Cpx was 0.55, which corresponds to an intermediate position on the scale used, with the possibility of F > 10%, this being the most common score for drugs [24]. Log P corresponds to the octanol/water partition coefficient, a parameter that is linearly related to its free solvation energy, where 1.10 was obtained for Cpx and 1.28 for **7a** [25]. Ciprofloxacin also obtained a solubility of −4.03, high gastrointestinal absorption, and a TPSA of 74.57 Å^2^, thus confirming its similarity to drugs. Regarding **7a**, a Log s of −1.32, high gastrointestinal absorption, TPSA of 63.57 Å^2^ were obtained, in addition to passing Lipinski’s rules and being classified as drug-like. The complete pharmacokinetic parameters obtained can be found in the Supplementary Material, Table S3.

**Figure 4:**
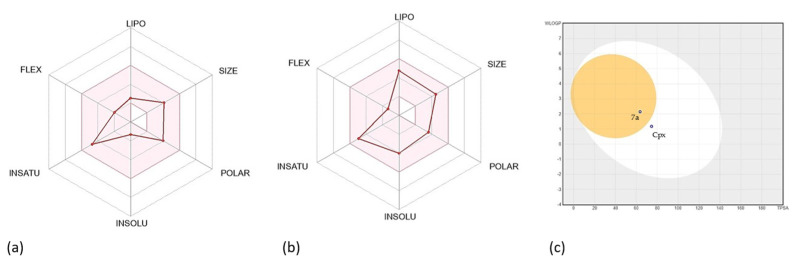
Bioavailability parameters of **7a** and CPX. (**a**) Radar bioavailability plot of Cpx. (**b**) Radar bioavailability plot of **7a**. (**c**) Boiled-Egg graph prediction for **7a** and Cpx. It is shown that both Cpx and **7a** are compounds with properties suitable for administration as oral drugs, and that **7a** would be capable of crossing the blood–brain barrier.

The bioavailability of the drug was predicted to be using radial bioavailability plots. Each axis represents a different parameter related to the oral bioavailability of the compounds, as seen in Figure 4a,b.

The boiled egg prediction model was also obtained, which was constructed using the topological surface area (TPSA) and lipophilicity (logP) of proposed compounds to analyze absorption in the gastrointestinal tract and penetration into the blood–brain barrier. The parameters that have been determined to cross the gastrointestinal barrier are in the white zone, while those that are also capable of crossing the blood–brain barrier are in the yellow zone (Figure 4c).

### 2.4. Compound ***7a*** Did Not Demonstrate Significant Toxicological Properties, Nor Did Its Predicted Metabolites

**Figure 5:**
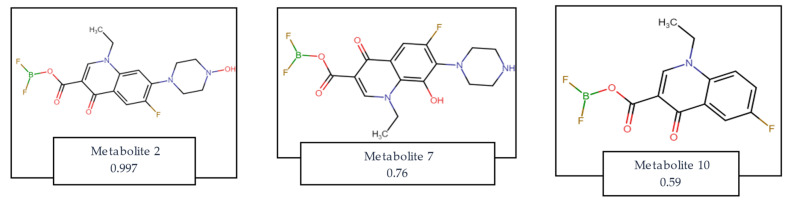
Structure of metabolites predicted on the Way2Drug platform for compound **7a**. The predicted probability coefficient for the formation of the compound after metabolism is added.

**Figure 6:**
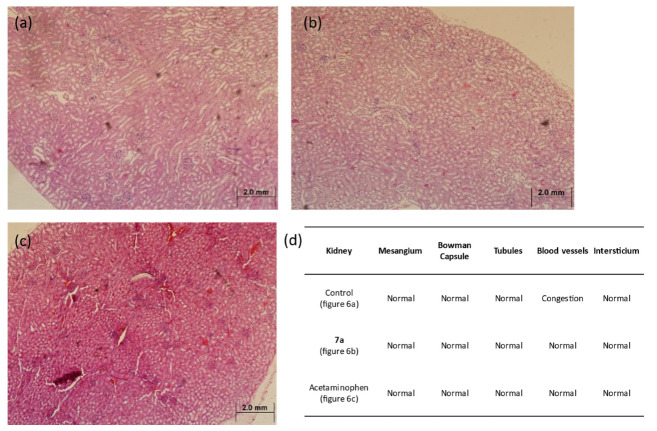
Kidney sections of mice model after acetaminophen administration. Representative images of H&E-stained renal tissue (4×) from Balb/c mice, *n* = 5 per experimental group. (**a**) Control mice without administration; (**b**) experimental group with administration of **7a**; (**c**) experimental group with administration of acetaminophen; (**d**) summary table of morphology analyzed in the tissue shown. None of the groups showed signs of nephrotoxicity.

Following the results presented above and predicting that compound **7a** would exhibit favorable pharmacological properties, we proceeded to evaluate the potential biological activity of its predicted metabolites, including possible adverse or toxic effects. Using the Way2Drug/MetaTox platform, the analysis indicated that the most probable metabolites would retain pharmacological characteristics consistent with antibacterial compounds (Table 4). Interestingly, the model also predicted a potential activity related to the treatment of Alzheimer’s disease for metabolite 10. Figure 5 displays the chemical structures of the metabolites with the highest predicted probability of formation, as determined by the MetaTox algorithm, along with their respective formation probability coefficients. Metabolite 2 would be formed after N-hydroxylation, metabolite 7 would be formed after aromatic hydroxylation, and metabolite 10 is predicted to be formed after N-dealkylation of compound **7a**. All the predicted metabolites can be found in Supplementary Material, Table S2:

Following the favorable predictive toxicity outcomes, an in vivo toxicity model was conducted. Histological examination of kidney tissues revealed no adverse alterations in any of the experimental groups, maintaining a normal renal morphology, as shown in Figure 6.

**Figure 7:**
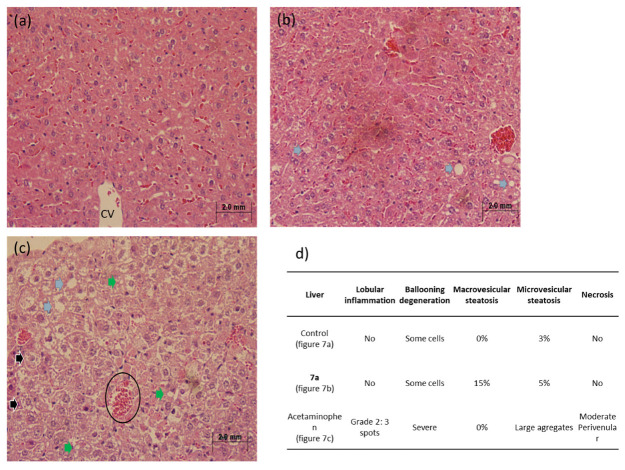
Liver sections of mice model after acetaminophen administration. Representative images of H&E-stained hepatic tissue (4×) from Balb/c mice, *n* = 5 per experimental group. (**a**) Control mice without administration; (**b**) experimental group with administration of **7a**; (**c**) experimental group with administration of acetaminophen; (**d**) summary table of morphology analyzed in the tissue shown. Clear evidence of hepatotoxicity is evident in the acetaminophen control group, with the morphological observations summarized. Black circle: cells infiltration; black arrows: ballooning degeneration; blue arrows: macrovesicular steatosis; green arrows: microvesicular steatosis.

Conversely, analysis of hepatic tissue demonstrated distinct results. As expected, the acetaminophen control group exhibited characteristic features of hepatotoxicity, including marked lobular inflammation, severe ballooning degeneration, extensive microvesicular steatosis, and moderate necrotic foci. In contrast, the **7a**-treated group showed only mild macrovesicular steatosis, a reversible change commonly observed under transient metabolic stress conditions (Figure 7).

**Figure 8:**
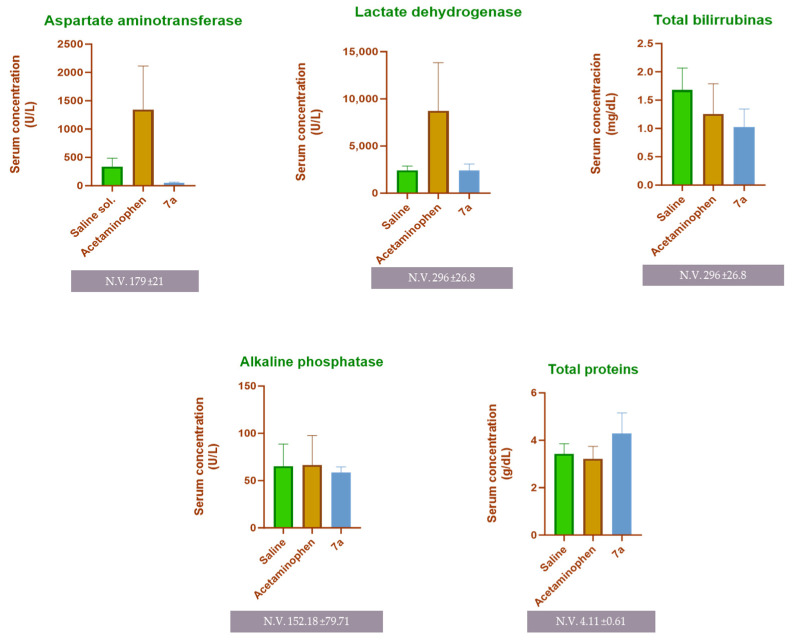
Serum biochemical analysis of mice subjected to the acute toxicity model. Mean and standard deviation 24 h after acute administration of the specified treatment are represented. The in vivo toxicity model was conducted using BALB/c mice (*n* = 5 per experimental group). One-way ANOVA and Tukey’s post-test were performed. The acetaminophen-treated group showed a significant increase in AST and LDH levels compared to control and **7a**-treated groups, while the rest of indicators remained unchanged, indicating no detectable renal or systemic toxicity (NV: normal values).

In addition to histopathological evaluation, serum samples collected from the experimental mice were analyzed for biochemical markers of hepatic and renal function. As shown in Figure 8, the acetaminophen-treated group exhibited a marked elevation in serum levels of AST (1347 U/L) and LDH (8718.54 U/L), indicating the hepatocellular damage expected from acute toxic exposure. Conversely, no significant differences were observed among the experimental groups for total bilirubin, alkaline phosphatase, or total protein levels, suggesting the absence of renal impairment and consistent with the acute nature of the model.

### 2.5. Compound ***7a*** Halted Pathogenic Progression in a Pneumonic Mice Model

Based on the previous findings, the evaluation progressed to an in vivo pneumonia model using the *S. aureus* strain with induced resistance. The infection was established with an inoculum of approximately 9 × 10^7^ CFU of *S. aureus*, consistent with previously reported concentrations that ensure biological survival and reproducibility of infection models [26,27]. Experimental conditions followed previously described protocols, employing a non-immunosuppressed infection route to avoid interference with host defense mechanisms [17,28]. Throughout the experiment, no significant variations were detected in the daily body weight of the treated groups, indicating overall tolerance to the procedure and treatments.

**Figure 9:**
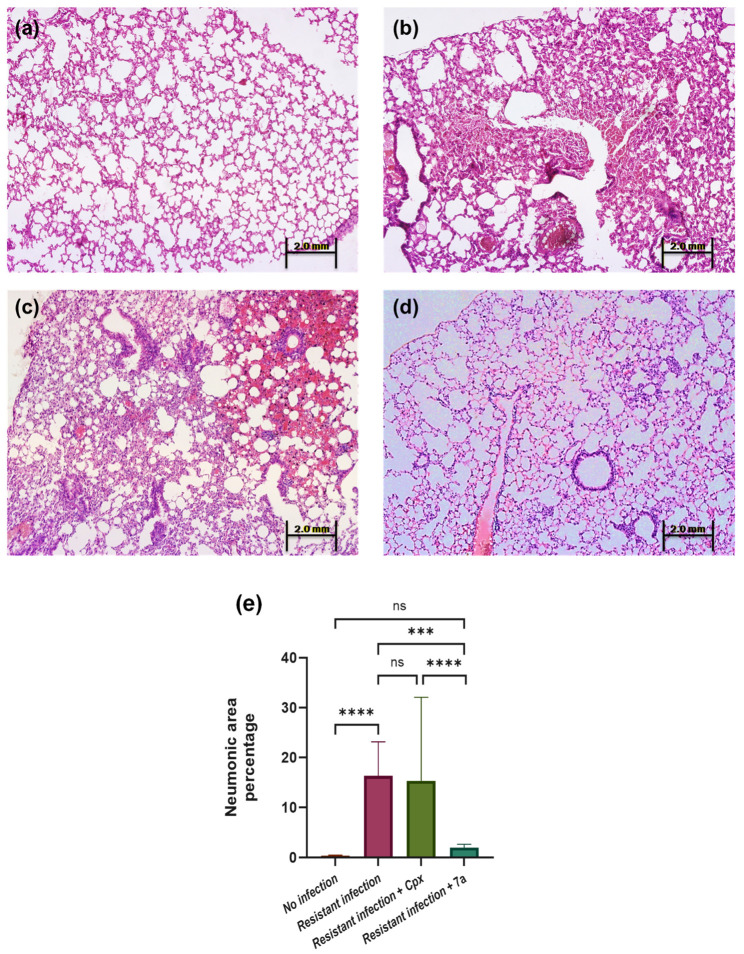
Histopathological evaluation of lung tissue sections from the murine pneumonia model. Representative images of H & E-stained lung tissue (4×) from Balb/c mice *n* = 5 per experimental group, infected with 9 × 10^7^ CFU of S. aureus intratracheally and treated during five days post-infection. (**a**) Normal lung histology of uninfected mice. (**b**) Untreated *S. aureus*-infected group. (**c**) Ciprofloxacin-treated resistant strain group. (**d**) Group treated with compound **7a**. (**e**) Comparative bar chart summarizing pneumonic area percentages across all experimental groups. Statistical analysis of one-way ANOVA and Tukey post-test were performed, where significant differences are expressed as follows: ns = not significant, *** *p* < 0.001, **** *p* < 0.0001.

Following tissue collection at the end of the in vivo pneumonia evaluation, whole-lung samples were examined, and twenty digital microscopic fields per mouse were analyzed. The mean pneumonic area percentage was calculated for each experimental group (*n* = 5). Representative histological images are shown in Figure 9a–d. As expected, healthy control lungs (Figure 9a) exhibited normal alveolar morphology, while the untreated *S. aureus*-infected group displayed extensive inflammatory infiltration, with an average pneumonic area of 16.32% (*p* < 0.05; Figure 9b).

In contrast, the ciprofloxacin-treated group (Figure 9c) showed a comparable level of pulmonary inflammation (15.30%), confirming that the antibiotic was ineffective against the resistant *S. aureus* strain. Remarkably, mice treated with compound **7a** exhibited a pronounced reduction in lung pathology, with only 1.99% of affected tissue—significantly lower than the infection control group (*p* < 0.001; 95% CI: 1.75–2.43; Figure 9d). Quantitative comparisons among the groups are summarized in Figure 9e, highlighting the therapeutic potential of **7a** in attenuating the progression of pneumonic lesions.

The results in the described model evidenced that compound **7a** halted pathogenic progression in a pneumonia model, unlike ciprofloxacin, which was not effective against the strain with induced resistance.

### 2.6. Compound ***7a*** Inhibits Bacterial DNA Gyrase in an Electrophoretic Assay

**Figure 10:**
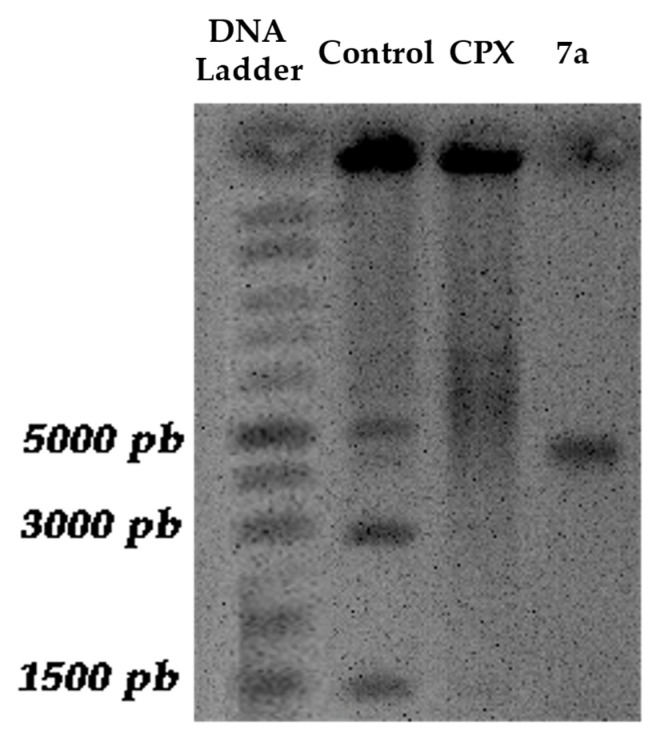
Agarose gel electrophoresis of plasmid DNA (5371 bp) extracted after DNA gyrase assay. DNA Ladder Lane: DNA molecular weight marker (1–10 kb ladder). Control Lane: untreated control showing the characteristic migration pattern of plasmid DNA, including the fast-migrating supercoiled form and the slower-migrating open circular (nicked) form. CPX Lane: ciprofloxacin-treated sample displaying accumulation of relaxed and nicked topoisomers, evidenced by reduced intensity of the supercoiled band and diffuse slower-migrating bands. **7a** Lane: **7a**-treated sample showing the disappearance of the two faster-migrating bands at 1500 bp and 3000 bp and the accumulation of the linear topoisomer, consistent with inhibition of DNA gyrase–mediated supercoiling.

After confirming the antimicrobial activity of compound **7a** against *Staphylococcus aureus*, a DNA gyrase inhibition assay was performed to determine whether its mechanism of action involved interference with DNA supercoiling, as described for fluoroquinolones. Supercoiled plasmid DNA (5371 bp) was incubated with the enzyme in the presence or absence of ciprofloxacin (Cpx) or compound **7a**. Reaction products were analyzed by agarose gel electrophoresis using a molecular weight marker covering the 1–10 kb range (Figure 10).

In the untreated control, the plasmid exhibited the characteristic migration pattern of negatively supercoiled DNA, appearing as three main bands (two faster-migrating bands at 1500 bp and 3000 bp), along with the linear band (5000 bp) and minor open circular (nicked) forms migrating more slowly (larger than 5000 bp). In contrast, samples treated with ciprofloxacin showed accumulation of relaxed and nicked topoisomers (larger than 5000 bp), reflected by a shift toward slower-migrating bands and a diffuse distribution pattern, consistent with stabilization of the DNA–gyrase cleavage complex.

Similarly, treatment with compound **7a** resulted in the disappearance of the two faster-migrating bands at 1500 bp and 3000 bp and the accumulation of the linear topoisomer. Importantly, the resulting band is approximately 5000 bp, suggesting that the differences in electrophoretic mobility were due to alterations in DNA topology rather than fragmentation. These results indicate that compound **7a** effectively inhibits DNA gyrase activity, supporting the molecular docking predictions and aligning with the established mechanism of action of fluoroquinolones [29,30].

The results obtained demonstrate the inhibitory capacity of compound **7a** on DNA gyrase, confirming previous results obtained in molecular docking evaluations in this research.

## 3. Discussion

The in silico results indicate that compound **7a** binds to the catalytic site of *S. aureus* DNA gyrase with an affinity comparable to or greater than that of ciprofloxacin, suggesting promising inhibitory potential. Furthermore, predicted interactions for **7a** establishes contacts with different functional groups in the binding pocket, which could allow it to maintain activity against variants of the enzyme that exhibit classic fluoroquinolone resistance mutations [31,32]. Such **7a** interaction would take place in the Gyr-A subunit, which is widely recognized as the catalytic site of the enzyme [21,33,34]. Taken together, these findings support the hypothesis that **7a** could offer a therapeutic advantage over conventional fluoroquinolones.

It is also worth noting the score of our compound **7a** (−10.1 kcal/mol), which predicts probable activity, with better affinity energy than other reported quinolone compounds, and which, having in vitro activity, yields docking evaluations ranging from −8.51 to −7.43 [22].

In vitro MIC and MBC assessments reveal the potency and effectiveness of compound **7a** against susceptible bacterial strains according to CLSI parameters, and as previously reported [17,19]; while continuing to show activity consistent with ciprofloxacin. This result suggests that structural modification maintains the inherent capacity of the fluoroquinolone class. Achieving an MIC of 0.25 µg/mL (on par with ciprofloxacin) against *S. aureus*, compound **7a** demonstrates a level of activity comparable to that of the latest-generation agents such as delafloxacin (MIC_90_ ≈ 1 µg/mL) [35]. Furthermore, recent medicinal chemistry efforts continue to achieve similar or lower MIC values (0.125–0.5 µg/mL) in novel quinolone analogues [36]. In other evaluations with fluoroquinolone derivatives, MICs as low as 0.04–6.25 µg/mL were reported, indicating that reduced MICs against susceptible strains of *S. aureus* represent a good starting point for candidate molecules [37].

Resistance has been reported to be generated in *S. aureus* strains after a consecutive reseeding with Sub-MIC antibiotic stimulus [38]. It has been evidenced that series of 25 passages can reach an elevated MIC of 120 µg/mL [39]. In contrast, in the experiment described here, we achieved high resistance to ciprofloxacin after 14 days of consecutive re-seeding. It is also noteworthy that, using the strain with the generated resistance, compound **7a** is able to continue inhibiting its development in vitro, which led to high expectations regarding the rest of its evaluations.

The in silico ADME/T profile of compound **7a** supports its potential as an orally administered antibacterial candidate [40]. SwissADME predictions indicate favorable drug-likeness and oral bioavailability, together with a propensity to cross the blood–brain barrier (BBB) and to act as a P-glycoprotein (P-gp) substrate, a combination that parallels several properties reported for ciprofloxacin and other quinolones [41]. While BBB permeability may favor central nervous system exposure, P-gp substrate status can limit net brain accumulation and should be considered when interpreting CNS–penetration predictions ([42,43]). Complementary MetaTox/Way2Drug analysis did not identify likely metabolites with high probabilities of severe toxicity; predicted biotransformations were consistent with retention of antibacterial scaffolding rather than generation of highly toxic species [44]. Taken together, these in silico data suggest that **7a** has a pharmacokinetic and safety profile comparable to established fluoroquinolones, supporting further in vitro and in vivo pharmacokinetic and toxicological validation [45].

With regard to toxicity, our compound **7a** showed favorable results in vivo, where after analyzing the collected tissues, normal morphology was observed in the kidneys and insignificant changes in the liver, unlike the pathological process evidenced by acetaminophen. Regarding the evaluation of serum markers, it is expected that a hepatotoxic process will generate an elevation of AST and LDH [46], denoting hepatocellular disease, as shown in the acetaminophen experimental group. However, the results of **7a** are equivalent to those of the control group without stimuli. The combination of elevated levels of the before mentioned biomarkers, without alterations in total proteins, total bilirubin, or alkaline phosphatase, can be explained by the short duration of an acute toxicity model, which would not be sufficient to cause damage to elevate them. In addition, no nephrotoxic effects were found that would have caused alterations in these parameters [47]. Mice treated with **7a** showed only mild macrovesicular steatosis and no necrosis or severe inflammation, findings that are considered reversible and less indicative of acute functional damage. Recovery and reversibility of steatosis after a single exposure are documented in preclinical studies, where the lack of simultaneous elevation of critical serum markers is usually correlated with transient processes [48].

Furthermore, the safety profile observed for **7a** is consistent with the experiences reported for compounds such as delafloxacin and finafloxacin. In single-dose and repeat-dose trials in rodents and dogs, the most prominent adverse effects are usually gastrointestinal and reversible tissue changes, with no direct mortality attributable to the drug [49]. Likewise, studies in murine models with finafloxacin have documented good efficacy and tolerability in acute treatments for respiratory infections without evidence of severe acute toxicity in histological and serological parameters, using ~30 mg/kg orally [50]. The results suggest that **7a** has an acute toxicological profile comparable to or more favorable than that of these reference fluoroquinolones in preclinical studies.

The in vivo pneumonia model using *Staphylococcus aureus* resistant to ciprofloxacin provided strong evidence of the therapeutic limitations of conventional fluoroquinolones and the promising activity of the novel compound **7a**. The slow progression of pneumonia in the group treated with **7a** indicates a significant protective effect and suppression of bacterial proliferation. These results suggest that **7a** retains antibacterial efficacy even against strains that have developed resistance to ciprofloxacin, consistent with its predicted higher binding affinity to DNA gyrase and favorable ADME properties. Comparable studies have demonstrated that fluoroquinolone resistance in *S. aureus* often arises from mutations in *gyrA* and *parC* genes, reducing ciprofloxacin binding, while novel quinolone derivatives with modified substituents can overcome this resistance by improving molecular interactions at the gyrase binding pocket [21,22,51,52].

Clinically important drugs such as delafloxacin have demonstrated a reduction in pulmonary bacteria and histological improvement in murine models of MRSA pneumonia, while maintaining adequate plasma levels. In comparison, compound **7a** maintains comparable efficacy and exhibits a better efficacy-to-toxicity ratio, considering its low incidence of adverse effects in vivo [49]. The aforementioned delafloxacin has also been evaluated in a *K. pneumoniae* pneumonia model, where it showed a 1 log bactericidal elimination relative to the initial bacterial load, but requiring 235 mg/kg/day administrations [53]. This demonstrates the efficacy of **7a** and its in vivo potency, as only 80 mg/kg/day was used to inhibit the progression of the resistant pneumonic process.

In support of these findings, agarose gel electrophoresis of plasmid DNA (5371 bp) recovered from *E. coli* DH5α cells exposed to compound **7a** or ciprofloxacin revealed alterations consistent with DNA gyrase inhibition. In the untreated control, plasmid DNA exhibited the characteristic migration pattern of negatively supercoiled DNA, with a predominant fast-migrating supercoiled band and a slower-migrating open circular (nicked) form. These conformational isoforms reflect the dynamic equilibrium of plasmid topology under normal gyrase activity.

Exposure to ciprofloxacin resulted in a marked reduction in the supercoiled form and the accumulation of slower-migrating relaxed and open circular topoisomers, accompanied in some cases by a faint linear form. This pattern is consistent with stabilization of the DNA–gyrase cleavage complex and impairment of DNA supercoiling. Similarly, treatment with compound **7a** produced the disappearance of the supercoiled band and enrichment of the linear topoisomer, without evidence of discrete low–molecular weight DNA fragments, indicating that the observed mobility shifts were due to topological changes rather than plasmid degradation.

Collectively, these findings support that compound **7a** interferes with DNA gyrase–mediated supercoiling in a manner comparable to ciprofloxacin, reinforcing the mechanistic hypothesis derived from molecular docking analyses. This behavior is consistent with the established mechanism of fluoroquinolones, which disrupt bacterial DNA topology by stabilizing gyrase–DNA cleavage intermediates and preventing religation of DNA strands, ultimately leading to replication arrest and cell death [54,55,56].

Overall, the present in vivo and in vitro findings reinforce the potential of compound **7a** as a next-generation quinolone capable of overcoming fluoroquinolone resistance in *S. aureus*. Its ability to significantly reduce pulmonary infection and inhibit DNA gyrase validates its dual antibacterial and mechanistic effectiveness, positioning it as a strong candidate for further preclinical development.

## 4. Materials and Methods

### 4.1. Synthesis of Difluoroboranyl 1-Ethyl-7-fluoro-4-oxo-7-piperazin-1-yl-1,4-dihydro-quinoline-3-carboxylate, ***7a***

The compound **7a**, a fluoroquinolone derived molecule designed to enhance pharmacological activity through forming a complex with boron [28] was synthesized according to the method described by Hernández-López et al. [57], with some modifications, as described in another work [17]. In a reflux system, 1.5 mL of acetonitrile (Sigma-Aldrich, St. Louis, MO, USA), 69.4 μL (0.5 mmol) of triethylamine (TEA) (Sigma-Aldrich, St. Louis, MO, USA), 100 mg (332.85 μmol) of difluoroboryl 1-ethyl-6,7-difluoro-4-oxo-1,4-dihydro-quinoline-3-carboxylate, and 43 mg (0.5 mmol) of piperazine (Sigma-Aldrich, St. Louis, MO, USA) were combined and heated at 80 °C for 10 h with continuous stirring. Afterwards, 1 mL of ethanol (Sigma-Aldrich, St. Louis, MO, USA) was added to the reaction mixture, yielding a light-yellow precipitate that was collected by vacuum filtration and washed repeatedly with ethanol. A yellow solid product was obtained, in 79% reaction yield, with a melting point of 230–231 °C (determined using a Fisher–Johns melting point apparatus (Thermo Fisher Scientific, Waltham, MA, USA)). The spectroscopy characterization of compound **7a** was consistent with reported data [15]: ^1^H NMR (Varian Mercury plus 400 MHz spectrometer using TMS as the internal control, DMSO-*d*_6_) δ (ppm): 9.25 (*s*, 1H), 8.05 (*d*, *J*_HF orto_ = 13.48 Hz, 2H), 7.31 (*d*, *J*_HF meta_ = 7.31 Hz, 2H), 4.82 (*c*, *J*_HH_ = 7.11 Hz, 2H), 3.38 (m, 4H), 2.89 (m, 4H), 1.46 (t, *J*_HH_ = 7.11 Hz, 3H).

### 4.2. Molecular Docking

In order to assess the potential interaction between the compound **7a** and the *S. aureus* DNA gyrase (described target of fluoroquinolones) a molecular docking evaluation was conducted [33]. The crystallized structure of *S. aureus* DNA gyrase co-crystallized with ciprofloxacin (PDB ID: 2xct, 3.35 Å) was obtained from RSCB Protein Data Bank (RCSB PDB, Research Collaboratory for Structural Bioinformatics, Rutgers University, Piscataway, NJ, USA).

Non-essential structural components to the protein, such as solvent or water molecules, were removed in UCSF Chimera (v1.16; University of California, San Francisco, CA, USA), conversely, the DNA double strand and the Mg^2+^ ion, were preserved. The energy minimization of the protein structure was performed with the PDB2PQR parameter set. The receptor protonation state was obtained using AutoDock Tools (v1.5.7; The Scripps Research Institute, La Jolla, CA, USA).

The ligand structure of compound **7a** was sketched in BIOBIA Draw (v19.1.0; Dassault Systèmes BIOVIA, San Diego, CA, USA) and later imported into Avogadro (v1.2.0; University of Pittsburgh, Pittsburgh, PA, USA) for geometry optimization using the UFF force field and the steepest descent algorithm (4 steps by update). The torsion degrees of the ligand and the grid box parameter for the docking zone were defined in AutoDock Tools (v1.5.7), covering the entire DNA gyrase subunit A.

Molecular docking studies were conducted using *AutoDock Vina* (v1.1.2) [58] with an exhaustiveness parameter of 16 to evaluate the binding affinity of compound **7a** toward the *S. aureus* DNA gyrase receptor (PDB ID: 2XCT). Ciprofloxacin was included as a reference ligand to validate the docking protocol and enable comparative analysis of binding interactions. The resulting protein–ligand complexes were examined using the Receptor–Ligand Interaction module in *Discovery Studio Visualizer* (v21.1.0; Dassault Systèmes BIOVIA, San Diego, CA, USA) to identify key residues and interaction types involved in ligand binding.

### 4.3. Minimum Inhibitory Concentration and Minimum Bactericidal Concentration

The Minimum Inhibitory Concentration (MIC) of compound **7a** was determined using the microdilution method in triplicate. Serial twofold dilutions ranging from 128 µg/mL to 0.125 µg/mL (except for the strain that was subsequently resistant to ciprofloxacin, which was evaluated up to 1024 μg/mL) were prepared in Müeller–Hinton (MH) broth (Bioxon, Cat. No. 210300, Becton Dickinson and Company, Querétaro, Mexico) within 96-well microplates. Each well was inoculated with a standardized bacterial suspension containing 5 × 10^4^ CFU/mL of *Staphylococcus aureus* (clinical isolate of Methicillin Resistant *Staphylococcus aureus* HGZ2201#ID), *Klebsiella pneumoniae*, or *Escherichia coli*; clinical isolates were obtained from the strain collection of the Universidad Autónoma de Zacatecas, where it was kept frozen at −80 °C until use; bacteria were originally obtained and donated from isolates of pathological infections in patients at the General Hospital of Zacatecas.

Microplates were incubated for 24 h at 37 °C under aerobic conditions, and bacterial growth was visually determined by the clear formation of a bacterial pellet. Growth inhibition was defined as the absence of turbidity and pellet formation.

For determination of the Minimum Bactericidal Concentration (MBC), aliquots from the three lowest concentrations showing no visible growth were reseeded onto Müeller–Hinton agar (MHA) (Bioxon, no. cat. 211667, Becton Dickinson and Company, Querétaro, Mexico) plates and incubated under the same conditions. The MBC was defined as the lowest concentration of compound **7a** at which no colony-forming units (CFU) were observed after 24 h of incubation at 37 °C.

As reference control, ciprofloxacin was simultaneously evaluated under identical conditions to confirm bacterial susceptibility and to provide a comparative benchmark for the bacteriostatic and bactericidal performance of compound **7a** [59,60].

### 4.4. Generation of Ciprofloxacin-Resistant S. aureus

The induction of ciprofloxacin-resistant *Staphylococcus aureus* was performed using a serial adaptive exposure approach. An aliquot of an overnight culture of *S. aureus* was inoculated into a 96-well microplate containing MH broth supplemented with subinhibitory concentrations of ciprofloxacin (below the previously established MIC) in triplicate. After 24 h of incubation at 37 °C under aerobic conditions, the visible bacterial pellet was transferred to fresh medium with the subinhibitory antibiotic concentration. This reseeding process was repeated daily for 14 consecutive passages. MIC of ciprofloxacin for the replated culture was reevaluated to confirm the development of resistance.

This methodology, based on gradual exposure to antibiotics, has been described as an effective strategy for generating resistant strains [38,39,61].

### 4.5. ADME Property and Metabolite Toxicity Simulation

The pharmacokinetic profiling of compound **7a** was conducted using the SwissADME web platform “https://www.swissadme.ch accessed on 22 March 2025” which allows in silico prediction of Absorption, Distribution, Metabolism, and Excretion (ADME) properties of small molecules based on their structural features [40]. The chemical structure of compound **7a** was drawn in BIOVIA Draw (version 19.1.0) and uploaded to the SwissADME interface in SMILES format for analysis. Parameters such as gastrointestinal absorption, blood–brain barrier permeability, P-glycoprotein substrate prediction, cytochrome P450 inhibition profile, and bioavailability score were calculated according to the implemented predictive algorithms (BOILED-Egg model and iLOGP) [41].

For comparative purposes, ciprofloxacin was included as a reference compound to assess the relative pharmacokinetic behavior between a clinically validated fluoroquinolone and compound **7a**. The simulation data were automatically generated and visualized through the SwissADME platform output.

The potential metabolic pathways and toxicity of compound **7a** were evaluated using the MetaTox web server, available at Way2Drug “https://way2drug.com/dr/ accessed on 12 April 2025”. This platform predicts phase I and phase II biotransformations based on a combination of structure–activity relationships and fragment-based descriptors, enabling estimation of possible metabolites and their toxicological profiles [44].

The structure of compound **7a**, previously optimized and converted to SMILES format, was uploaded into the MetaTox interface. The algorithm simulated oxidation, reduction, and conjugation reactions catalyzed by cytochrome P450 isoenzymes (CYP450s) and other metabolic systems. Each predicted metabolite was subsequently analyzed for its potential mutagenic, carcinogenic, and hepatotoxic risks using the integrated toxicity assessment modules.

Ciprofloxacin was also analyzed as a control to assess whether the boron-containing fluoroquinolone **7a** analog exhibits similar or improved metabolic stability and safety. The results were exported, and images of the predicted metabolites were also obtained.

### 4.6. In Vivo Toxicity Mice Model

A murine model of acute toxicity was implemented using Balb/c mice of both sexes, kept under standard animal facility conditions (12 h light/dark cycle, controlled temperature of 25 °C, and free access to water and food). The animals were divided into three experimental groups (*n* = 5 per group). The negative control group received only isotonic saline solution (0.9% NaCl) intraperitoneally. The positive control group was treated with acetaminophen at a dose of 300 mg/kg to induce a model of acute hepatotoxicity. Finally, the experimental group received a single dose of 300 mg/kg of compound **7a**, administered by the same route, to evaluate a possible hepatotoxic and/or nephrotoxic effect [62,63,64].

Twenty-four hours after administration, the animals were euthanized by cardiac puncture under deep anesthesia, and whole blood serum was collected for subsequent biochemical analysis. The liver and kidneys were dissected, fixed in 10% neutral formalin, and processed for paraffin embedding. Histological sections of 5 µm were obtained and stained with hematoxylin and eosin (H&E) for microscopic evaluation. The serum was used for colorimetric quantification of biomarkers of liver and kidney damage [65]. Biochemical parameters including aspartate aminotransferase (AST), lactate dehydrogenase (LDH), total bilirubin, alkaline phosphatase (ALP), and total proteins were quantified using the Ortho Clinical Vitros 250 Chemistry System (© Ortho-Clinical Diagnostics, Inc., Raritan, NJ, USA), following the laboratory’s internal quality control protocols.

### 4.7. Acute Pneumonia In Vivo Model

**Figure 11:**
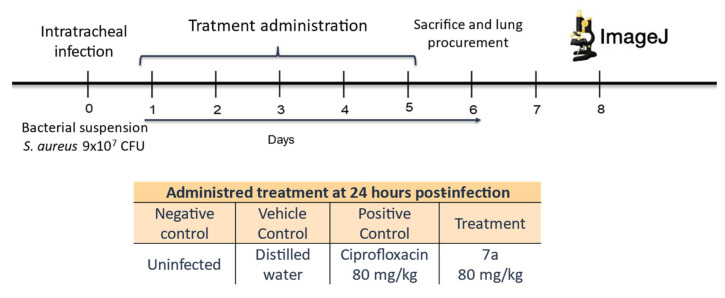
Experimental treatment design of intratracheal resistant infection. Bacterial inoculation was performed on day zero, with 9 × 10^7^ CFU of *S. aureus*. Treatments and controls were administered 24 h after infection for 5 consecutive days. On day 6, the animals were sacrificed, and their lungs were dissected. The orange-highlighted header identifies the experimental groups, each assigned the treatment specified in the unshaded cells, including the uninfected control, and infected groups treated with injectable water, ciprofloxacin, or compound **7a**.

The murine model of acute bacterial pneumonia was established using sixteen BALB/c mice (male and female, 10–12 weeks of age), consistent with previously validated protocols for respiratory infection models [66,67]), and were assigned to four experimental groups (*n* = 5 each, Figure 11): (i) uninfected control, (ii) *S. aureus*-infected untreated, (iii) *S. aureus*-infected treated with ciprofloxacin, and (iv) *S. aureus*-infected treated with compound **7a**. All bacterial infections were performed with the ciprofloxacin-resistant MRSA strain mentioned above. Mice were maintained under pathogen-free conditions with a 12 h light/dark cycle and had ad libitum access to sterile food and water. All experimental procedures complied with the Mexican regulation NOM-062-ZOO-1999 for laboratory animal care and use and were approved by the Institutional Ethics Committee of the Autonomous University of Zacatecas (protocol ID: SACS/UAZ/308/2020).

For infection, animals were anesthetized with sodium pentobarbital (1.5 mL/5 kg, i.p.; Cheminova, cat. no. 30375-B, Cheminova, Lemvig, Denmark) and intratracheally inoculated with 50 μL of a *S. aureus* suspension containing a total of 9 × 10^7^ CFU in sterile saline, following established pulmonary infection models [68]. Treatments began 24 h post-infection via intraperitoneal injection: compound **7a** at 80 mg/kg/day for five consecutive days, or ciprofloxacin at the same dose, consistent with previous pharmacodynamic assessments of fluoroquinolones in rodent models [69,70]. Body weight was recorded daily to monitor systemic tolerance [71].

On day six, mice were euthanized by CO_2_ inhalation, and lungs were perfused with 10% formalin via the trachea to preserve tissue morphology [72]. The right lung from each animal was fixed, paraffin-embedded, and sectioned (2 μm). Histological sections were stained with hematoxylin and eosin (H&E) and examined at 40× magnification using an Olympus^®^ inverted microscope [67,68,72]. Digital micrographs were analyzed with ImageJ software (v1.52) to quantify pneumonic areas. Using the polygon tool, alveolar regions showing consolidation and inflammatory cells infiltration were delineated, and the total pneumonic area (cm^2^) was expressed as a percentage of the total lung tissue [27,73].

### 4.8. DNA Gyrase Activity Inhibition Test on the Supercoiling of the pGLO Plasmid

To evaluate whether compound **7a** interferes with DNA supercoiling in a manner consistent with fluoroquinolone activity, a cellular assay was performed using *Escherichia coli* DH5-α cells transformed with the pGLO plasmid (5371 bp). Under physiological conditions, negative supercoiling of plasmid DNA is maintained by endogenous bacterial DNA gyrase activity [74].

Three experimental groups were established from exponentially growing cultures (OD_600_ ≈ 0.8): (1) untreated control cells grown in LB medium supplemented with ampicillin (100 µg/mL) to maintain plasmid selection; (2) cells exposed to ciprofloxacin (1 µg/mL) for 4 h at 37 °C; and (3) cells treated with compound **7a** (1 µg/mL) for 4 h at 37 °C.

Drug concentrations were selected based on antibacterial susceptibility assays and maintained under conditions that preserved cell viability during the exposure period.

Following treatment, plasmid DNA was isolated using the alkaline lysis miniprep method [75,76]. DNA samples were mixed with 6× loading dye and subjected to electrophoresis on 1% agarose gels in TAE buffer at 80 V for 45 min. Gels were stained with ethidium bromide and visualized under UV illumination using a DNR MiniBIS Pro imaging system. Migration patterns corresponding to supercoiled, relaxed (open circular), and linear topoisomers were qualitatively compared between groups. Alterations in the distribution of plasmid conformations were interpreted as indirect evidence of interference with DNA gyrase–mediated supercoiling, consistent with previously described quinolone–gyrase interaction models [29,77].

### 4.9. Statistical Analysis

All experimental data were initially subjected to a normality assessment using the Shapiro–Wilk test. Datasets exhibiting normal distribution were analyzed by one-way analysis of variance (ANOVA) followed by Tukey’s multiple comparison post hoc test to determine significant differences between experimental groups. A significance level of *p* ≤ 0.05 was considered statistically significant. Data are expressed as mean ± standard deviation (SD), while mode values were used in categorical concentration comparisons. All analyses and graphical representations were performed using GraphPad Prism software (version 8.0.2).

## 5. Conclusions

The findings presented in this study demonstrate the efficacy and functional potential of compound **7a** against clinically relevant bacteria, including ciprofloxacin-resistant *S. aureus*. Through in vitro assays, compound **7a** exhibited potent antibacterial activity and a favorable toxicological profile, supported by predicted pharmacokinetic properties consistent with those required for orally administered drugs. Moreover, both in vitro and in vivo evaluations confirmed its ability to inhibit the proliferation of resistant *S. aureus* strains. Molecular docking and mechanistic assays further verified the inhibition of DNA gyrase, aligning with the expected mechanism of action of fluoroquinolones. Altogether, these results highlight compound **7a** as a promising candidate for future antibacterial drug development and support continued exploration of structurally related quinolone derivatives.

## Figures and Tables

**Table 1:** Affinity scores obtained in molecular docking.

Conformation Mode	7a Affinity (kcal/mol)	Ciprofloxacin Affinity (kcal/mol)
1	−10.0	−8.9
2	−9.9	−8.7
3	−9.8	−8.7
4	−9.5	−8.7
5	−9.5	−8.5
6	−9.4	−8.5
7	−9.3	−8.3
8	−9.3	−8.3
9	−9.1	−8.2

**Table 2:** MIC and MBC results for **7a** against susceptible strains.

*Bacterial Strain*	Compound	MIC (μg/mL)	MBC (μg/mL)
*S. aureus*	Ciprofloxacin	0.25	0.5
	**7a**	0.25	0.25
*K. pneumoniae*	Ciprofloxacin	1	2
	**7a**	1	1
*E. coli*	Ciprofloxacin	2	8
	**7a**	4	8

**Table 3:** Pharmacokinetic parameters obtained by SwissADME.

Compound	Ciprofloxacin	7a
**Biodisponibility**	0.55	0.55
**Log P**	1.10	1.28
**Log S (ESOL)**	−4.03	−1.32
**GI absorption**	High	High
TPSA * (Å^2^)	74.57 Å^2^	63.57 Å^2^
**Drug likeness (Lipinsky rule approved)**	Yes	Yes

**Table 4:** Potential activity of metabolites with the highest likelihood of formation.

7a Metabolite Activity	Metabolite Number
Apoptosis agonist	10
Treatment for Alzheimer’s disease, anti-amyloidogenic	10
DNA synthesis inhibitor	7
Antibacterial, cell wall synthesis inhibitor	2

## Data Availability

All data generated or analyzed during this study are included in this article and its Supplementary Material Files. Further enquiries can be directed to the corresponding author.

## Supplementary Materials

The following supporting information can be downloaded at: https://www.mdpi.com/article/10.3390/molecules31061044/s1, Figure S1: Hydrophobic surface of position 1 of **7a** in the interaction pocket; Table S1: Most probable interaction positions with *S. aureus* DNA gyrase through molecular docking; Table S2: Predicted metabolites of **7a** through Way2drug platform; Table S3: Pharmacokinetic parameters of **7a** simulated using the SwissAMDE platform.

## References

[1] Mohr K.I. (2016). History of Antibiotics Research. Curr. Top. Microbiol. Immunol..

[2] Ashfield T., Cooray M., Jimenez-Acha I., Riaz Z., Gifford D.R., Lagator M. (2025). Reflecting on Fleming’s caveat: The impact of stakeholder decision-making on antimicrobial resistance evolution. Microbiology.

[3] Hansson K., Brenthel A. (2022). Imagining a post-antibiotic era: A cultural analysis of crisis and antibiotic resistance. Med. Humanit..

[4] Yekani M., Azargun R., Sharifi S., Nabizadeh E., Nahand J.S., Ansari N.K., Memar M.Y., Soki J. (2023). Collateral sensitivity: An evolutionary trade-off between antibiotic resistance mechanisms, attractive for dealing with drug-resistance crisis. Health Sci. Rep..

[5] Agyeman W.Y., Bisht A., Gopinath A., Cheema A.H., Chaludiya K., Khalid M., Nwosu M., Konka S., Khan S. (2022). A Systematic Review of Antibiotic Resistance Trends and Treatment Options for Hospital-Acquired Multidrug-Resistant Infections. Cureus.

[6] Belachew S.A., Hall L., Erku D.A., Selvey L.A. (2021). No prescription? No problem: Drivers of non-prescribed sale of antibiotics among community drug retail outlets in low and middle income countries: A systematic review of qualitative studies. BMC Public Health.

[7] Salam M.A., Al-Amin M.Y., Salam M.T., Pawar J.S., Akhter N., Rabaan A.A., Alqumber M.A.A. (2023). Antimicrobial Resistance: A Growing Serious Threat for Global Public Health. Healthcare.

[8] Cassini A., Högberg L.D., Plachouras D., Quattrocchi A., Hoxha A., Simonsen G.S., Colomb-Cotinat M., Kretzschmar M.E., Devleesschauwer B., Cecchini M. (2019). Attributable deaths and disability-adjusted life-years caused by infections with antibiotic-resistant bacteria in the EU and the European Economic Area in 2015: A population-level modelling analysis. Lancet Infect. Dis..

[9] Sirota M., Juanchich M. (2024). Seeing an apocalyptic post-antibiotic future lowers antibiotics expectations and requests. Commun. Med..

[10] Murray C.J.L., Ikuta K.S., Sharara F., Swetschinski L., Robles Aguilar G., Gray A., Han C., Bisignano C., Rao P., Wool E. (2022). Global burden of bacterial antimicrobial resistance in 2019: A systematic analysis. Lancet.

[11] WHO (2025). Global Antibiotic Resistance Surveillance Report 2025. https://www.who.int/publications/i/item/9789240116337.

[12] Turner N.A., Sharma-Kuinkel B.K., Maskarinec S.A., Eichenberger E.M., Shah P.P., Carugati M., Holland T.L., Fowler V.G. (2019). Methicillin-resistant Staphylococcus aureus: An overview of basic and clinical research. Nat. Rev. Microbiol..

[13] Dine I., Mulugeta E., Melaku Y., Belete M. (2023). Recent advances in the synthesis of pharmaceutically active 4-quinolone and its analogues: A review. RSC Adv..

[14] Cormier R., Burda W.N., Harrington L., Edlinger J., Kodigepalli K.M., Thomas J., Kapolka R., Roma G., Anderson B.E., Turos E. (2012). Studies on the antimicrobial properties of N-acylated ciprofloxacins. Bioorganic Med. Chem. Lett..

[15] Leyva S., Hernández H. (2010). Synthesis of norfloxacin analogues catalyzed by Lewis and Brönsted acids: An alternative pathway. J. Fluor. Chem..

[16] Sayin K., Karakaş D. (2018). Investigation of structural, electronic properties and docking calculations of some boron complexes with norfloxacin: A computational research. Spectrochim. Acta Par. A Mol. Biomol. Spectrosc..

[17] Veyna-Hurtado L.A., Hernández-López H., Reyes-Escobedo F.d.R., de Loera D., García-Cruz S., Troncoso-Vázquez L., Galván-Valencia M., Castañeda-Delgado J.E., Cervantes-Villagrana A.R. (2025). The Derivative Difluoroboranyl-Fluoroquinolone “7a” Generates Effective Inhibition Against the S. aureus Strain in a Murine Model of Acute Pneumonia. Curr. Issues Mol. Biol..

[18] Medellín-Luna M.F., Hernández-López H., Castañeda-Delgado J.E., Martinez-Gutierrez F., Lara-Ramírez E., Espinoza-Rodríguez J.J., García-Cruz S., Portales-Pérez D.P., Cervantes-Villagrana A.R. (2023). Fluoroquinolone Analogs, SAR Analysis, and the Antimicrobial Evaluation of 7-Benzimidazol-1-yl-fluoroquinolone in In Vitro, In Silico, and In Vivo Models. Molecules.

[19] Veyna-Hurtado L.A., Hernández-López H., Reyes-Escobedo F., Medellín-Luna M., García-Cruz S., Troncoso-Vázquez L., González-Curiel I.E., Galván-Valencia M., Castañeda-Delgado J.E., Cervantes-Villagrana A.R. (2023). The difluoroboranyl-norfloxacin complex “7a” induces an antimicrobial effect against K. pneumoniae strain in acute pneumonia murine model. Med. Drug Discov..

[20] Kawsar S.M.A., Munia N.S., Saha S., Ozeki Y. (2024). In Silico Pharmacokinetics, Molecular Docking and Molecular Dynamics Simulation Studies of Nucleoside Analogs for Drug Discovery—A Mini Review. Mini Rev. Med. Chem..

[21] Norouzbahari M., Salarinejad S., Güran M., Şanlıtürk G., Emamgholipour Z., Bijanzadeh H.R., Toolabi M., Foroumadi A. (2020). Design, synthesis, molecular docking study, and antibacterial evaluation of some new fluoroquinolone analogues bearing a quinazolinone moiety. J. Fac. Pharm. Tehran Univ. Med. Sci..

[22] Patel M.M., Patel L.J. (2014). Design, synthesis, molecular docking, and antibacterial evaluation of some novel flouroquinolone derivatives as potent antibacterial agent. Sci. World J..

[23] (2020). Performance Standards for Antimicrobial Susceptibility Testing, 30th Edition.

[24] Martin Y.C. (2005). A bioavailability score. J. Med. Chem..

[25] Daina A., Michielin O., Zoete V. (2014). iLOGP: A Simple, Robust, and Efficient Description of n-Octanol/Water Partition Coefficient for Drug Design Using the GB/SA Approach. J. Chem. Inf. Model..

[26] Kim H.K., Missiakas D., Schneewind O. (2014). Mouse models for infectious diseases caused by Staphylococcus aureus. J. Immunol. Methods.

[27] Hraiech S., Papazian L., Rolain J.-M., Bregeon F. (2015). Animal models of polymicrobial pneumonia. Drug Des. Devel Ther..

[28] Arrazuria R., Kerscher B., Huber K.E., Hoover J.L., Lundberg C.V., Hansen J.U., Sordello S., Renard S., Aranzana-Climent V., Hughes D. (2022). Variability of murine bacterial pneumonia models used to evaluate antimicrobial agents. Front. Microbiol..

[29] Aleixandre V., Herrera G., Urios A., Blanco M. (1991). Effects of ciprofloxacin on plasmid DNA supercoiling of Escherichia coli topoisomerase I and gyrase mutants. Antimicrob. Agents Chemother..

[30] Han J., Wang Y., Sahin O., Shen Z., Guo B., Shen J., Zhang Q. (2012). A Fluoroquinolone Resistance Associated Mutation in gyrA Affects DNA Supercoiling in Campylobacter jejuni. Front. Cell. Infect. Microbiol..

[31] Phillips-Jones M.K., Harding S.E. (2018). Antimicrobial resistance (AMR) nanomachines—Mechanisms for fluoroquinolone and glycopeptide recognition, efflux and/or deactivation. Biophys. Rev..

[32] Huynh T.Q., Tran V.N., Thai V.C., Nguyen H.A., Nguyen N.T.G., Tran M.K., Nguyen T.P.T., Le C.A., Ho L.T.N., Surian N.U. (2023). Genomic alterations involved in fluoroquinolone resistance development in *Staphylococcus aureus*. PLoS ONE.

[33] Eberhardt J., Santos-Martins D., Tillack A.F., Forli S. (2021). AutoDock Vina 1.2.0: New Docking Methods, Expanded Force Field, and Python Bindings. J. Chem. Inf. Model..

[34] Hirsch J., Klostermeier D. (2021). What makes a type IIA topoisomerase a gyrase or a Topo IV?. Nucleic Acids Res..

[35] Ribeiro Á.C.d.S., Santos F.F., Valiatti T.B., Lenzi M.H., Santos I.N.M., Neves R.F.B., Moses I.B., Meneses J.P.d., Di Sessa R.G.d.G., Salles M.J. (2024). Comparative in vitro activity of Delafloxacin and other antimicrobials against isolates from patients with acute bacterial skin, skin-structure infection and osteomyelitis. Braz. J. Infect. Dis..

[36] Ommi O., Dhopat P.S., Sau S., Estharla M.R., Nanduri S., Kalia N.P., Yaddanapudi V.M.J.R.M.C. (2025). Design, synthesis, and biological evaluation of pyrazole–ciprofloxacin hybrids as antibacterial and antibiofilm agents against *Staphylococcus aureus*. RSC Med. Chem..

[37] Leyva-Ramos S., de Loera D., Cardoso-Ortiz J. (2017). In vitro Antibacterial Activity of 7-Substituted-6-Fluoroquinolone and 7-Substituted-6,8-Difluoroquinolone Derivatives. Chemotherapy.

[38] Sandegren L. (2014). Selection of antibiotic resistance at very low antibiotic concentrations. Upsala J. Med. Sci..

[39] Yasir M., Dutta D., Willcox M.D.P. (2021). Enhancement of Antibiofilm Activity of Ciprofloxacin against *Staphylococcus aureus* by Administration of Antimicrobial Peptides. Antibiotics.

[40] Daina A., Michielin O., Zoete V. (2017). SwissADME: A free web tool to evaluate pharmacokinetics, drug-likeness and medicinal chemistry friendliness of small molecules. Sci. Rep..

[41] Daina A., Zoete V. (2016). A BOILED-Egg To Predict Gastrointestinal Absorption and Brain Penetration of Small Molecules. ChemMedChem.

[42] Park M.S., Okochi H., Benet L.Z. (2011). Is Ciprofloxacin a Substrate of P-glycoprotein?. Arch. Drug Inf..

[43] Nau R., Sörgel F., Eiffert H. (2010). Penetration of drugs through the blood-cerebrospinal fluid/blood-brain barrier for treatment of central nervous system infections. Clin. Microbiol. Rev..

[44] Rudik A.V., Dmitriev A.V., Lagunin A.A., Filimonov D.A., Poroikov V.V. (2023). MetaTox 2.0: Estimating the Biological Activity Spectra of Drug-like Compounds Taking into Account Probable Biotransformations. ACS Omega.

[45] Jakobsen L., Lundberg C.V., Frimodt-Møller N. (2020). Ciprofloxacin Pharmacokinetics/Pharmacodynamics against Susceptible and Low-Level Resistant Escherichia coli Isolates in an Experimental Ascending Urinary Tract Infection Model in Mice. Antimicrob. Agents Chemother..

[46] Lala V., Zubair M., Minter D. Liver Function Tests. StatPearls 30 July 2023. https://www.ncbi.nlm.nih.gov/books/NBK482489/.

[47] Cancino K., Castro I., Yauri C., Jullian V., Arévalo J., Sauvain M., Adaui V., Castillo D. (2021). Evaluación de la toxicidad de chalconas sintéticas con potencial anti-Leishmania en ratones BALB/c. J. Rev. Peru. Med. Exp. Salud Publica.

[48] Muhammad-Azam F., Nur-Fazila S.H., Ain-Fatin R., Mustapha Noordin M., Yimer N. (2019). Histopathological changes of acetaminophen-induced liver injury and subsequent liver regeneration in BALB/C and ICR mice. Vet. World.

[49] Lodise T., Corey R., Hooper D., Cammarata S. (2018). Safety of Delafloxacin: Focus on Adverse Events of Special Interest. Open Forum Infect. Dis..

[50] Hartley M.G., Norville I.H., Richards M.I., Barnes K.B., Bewley K.R., Vipond J., Rayner E., Vente A., Armstrong S.J., Harding S.V. (2021). Finafloxacin, a Novel Fluoroquinolone, Reduces the Clinical Signs of Infection and Pathology in a Mouse Model of Q Fever. Front. Microbiol..

[51] Aldred K.J., Kerns R.J., Osheroff N. (2014). Mechanism of quinolone action and resistance. Biochemistry.

[52] Hooper D.C., Jacoby G.A. (2016). Topoisomerase Inhibitors: Fluoroquinolone Mechanisms of Action and Resistance. Cold Spring Harb. Perspect. Med..

[53] Zhao M., Lepak A.J., Marchillo K., Andes D.R. (2019). In Vivo Pharmacodynamic Target Determination for Delafloxacin against Klebsiella pneumoniae and *Pseudomonas aeruginosa* in the Neutropenic Murine Pneumonia Model. Antimicrob. Agents Chemother..

[54] Laponogov I., Sohi M.K., Veselkov D.A., Pan X.S., Sawhney R., Thompson A.W., McAuley K.E., Fisher L.M., Sanderson M.R. (2009). Structural insight into the quinolone-DNA cleavage complex of type IIA topoisomerases. Nat. Struct. Mol. Biol..

[55] Bush N.G., Diez-Santos I., Abbott L.R., Maxwell A. (2020). Quinolones: Mechanism, Lethality and Their Contributions to Antibiotic Resistance. Molecules.

[56] Blower T.R., Williamson B.H., Kerns R.J., Berger J.M. (2016). Crystal structure and stability of gyrase; fluoroquinolone cleaved complexes from Mycobacterium tuberculosis. Proc. Natl. Acad. Sci. USA.

[57] Hernández-López H., Sánchez-Miranda G., Araujo-Huitrado J.G., Granados-López A.J., López J.A., Leyva-Ramos S., Chacón-García L. (2019). Synthesis of Hybrid Fluoroquinolone-Boron Complexes and Their Evaluation in Cervical Cancer Cell Lines. J. Chem..

[58] Trott O., Olson A.J. (2010). AutoDock Vina: Improving the speed and accuracy of docking with a new scoring function, efficient optimization, and multithreading. J. Comput. Chem..

[59] Wiegand I., Hilpert K., Hancock R.E.W. (2008). Agar and broth dilution methods to determine the minimal inhibitory concentration (MIC) of antimicrobial substances. Nat. Protoc..

[60] Roszkowski P., Bielenica A., Stefańska J., Majewska A., Markowska K., Pituch H., Koliński M., Kmiecik S., Chrzanowska A., Struga M. (2024). Antibacterial and anti-biofilm activities of new fluoroquinolone derivatives coupled with nitrogen-based heterocycles. Biomed. Pharmacother..

[61] Toprak E., Veres A., Michel J.-B., Chait R., Hartl D.L., Kishony R. (2012). Evolutionary paths to antibiotic resistance under dynamically sustained drug selection. Nat. Genet..

[62] Mossanen J.C., Tacke F. (2015). Acetaminophen-induced acute liver injury in mice. Lab. Anim..

[63] Cayuela N., Koike M., Jacysyn J., Rasslan R., Cerqueira A., Costa S., Diniz J.A., Utiyama E., Montero E. (2020). N-Acetylcysteine Reduced Ischemia and Reperfusion Damage Associated with Steatohepatitis in Mice. Int. J. Mol. Sci..

[64] Jaeschke H., McGill M.R., Ramachandran A. (2012). Oxidant stress, mitochondria, and cell death mechanisms in drug-induced liver injury: Lessons learned from acetaminophen hepatotoxicity. Drug Metab. Rev..

[65] Shen X.L., Guo Y.N., Lu M.H., Ding K.N., Liang S.S., Mou R.W., Yuan S., He Y.M., Tang L.P. (2023). Acetaminophen-induced hepatotoxicity predominantly via inhibiting Nrf2 antioxidative pathway and activating TLR4-NF-κB-MAPK inflammatory response in mice. Ecotoxicol. Environ. Saf..

[66] Dietert K., Gutbier B., Wienhold S.M., Reppe K., Jiang X., Yao L., Chaput C., Naujoks J., Brack M., Kupke A. (2017). Spectrum of pathogen- and model-specific histopathologies in mouse models of acute pneumonia. PLoS ONE.

[67] Draxler D.F., Awad M.M., Hanafi G., Daglas M., Ho H., Keragala C., Galle A., Roquilly A., Lyras D., Sashindranath M. (2019). Tranexamic Acid Influences the Immune Response, but not Bacterial Clearance in a Model of Post-Traumatic Brain Injury Pneumonia. J. Neurotrauma.

[68] Morton D.B., Jennings M., Buckwell A., Ewbank R., Godfrey C., Holgate B., Inglis I., James R., Page C., Sharman I. (2001). Refining procedures for the administration of substances. Lab. Anim..

[69] Rodríguez-Martínez J.M., Pichardo C., García I., Pachón-Ibañez M.E., Docobo-Pérez F., Pascual A., Pachón J., Martínez-Martínez L. (2008). Activity of ciprofloxacin and levofloxacin in experimental pneumonia caused by *Klebsiella pneumoniae* deficient in porins, expressing active efflux and producing QnrA1. Clin. Microbiol. Infect..

[70] Thadepalli H., Bansal M.B., Rao B., See R., Chuah S.K., Marshall R., Dhawan V.K. (1988). Ciprofloxacin: In vitro, experimental, and clinical evaluation. Rev. Infect. Dis..

[71] Yamashita Y., Nagaoka K., Kimura H., Suzuki M., Konno S., Fukumoto T., Akizawa K., Kaku N., Morinaga Y., Yanagihara K. (2019). Efficacy of Azithromycin in a Mouse Pneumonia Model against Hospital-Acquired Methicillin-Resistant *Staphylococcus aureus*. Antimicrob. Agents Chemother..

[72] Aeffner F., Bolon B., Davis I.C. (2015). Mouse Models of Acute Respiratory Distress Syndrome: A Review of Analytical Approaches, Pathologic Features, and Common Measurements. Toxicol. Pathol..

[73] Mizgerd J.P., Skerrett S.J. (2008). Animal models of human pneumonia. Am. J. Physiol. Cell. Mol. Physiol..

[74] Jung S.C., Smith C.L., Lee K.S., Hong M.E., Kweon D.H., Stephanopoulos G., Jin Y.S. (2010). Restoration of growth phenotypes of Escherichia coli DH5alpha in minimal media through reversal of a point mutation in purB. Appl. Environ. Microbiol..

[75] Birnboim H.C., Doly J. (1979). A rapid alkaline extraction procedure for screening recombinant plasmid DNA. Nucleic Acids Res..

[76] Elnagar M.A., Ibrahim M.F., Albert M., Talal M.M., Abdelfattah M.M., El-Dabaa E., Helwa R. (2022). Homemade plasmid Miniprep solutions for affordable research in low-fund laboratories. AMB Express.

[77] Morgan-Linnell S.K., Hiasa H., Zechiedrich L., Nitiss J.L. (2007). Assessing sensitivity to antibacterial topoisomerase II inhibitors. Curr. Protoc. Pharmacol..

