# Supplementary material for: The Difluoroboranyl-Fluoroquinolone Derivative “7a” Inhibits Bacterial DNA Gyrase and Exhibits Potent Activity Against Ciprofloxacin-Resistant S. aureus In Vitro and In Vivo Using an Acute Pneumonia Model
Source: Molecules. 2026 Mar 20;31(6):1044. doi: 10.3390/molecules31061044 (PMC13029244; doi:10.3390/molecules31061044)
Supplement: Supplementary file 1 [file molecules-31-01044-s001.zip › molecules-4126464-supplementary.pdf]

Supplementary Materials

The difluoroboranyl- Fluoroquinolone derivative "7a" inhibits bacterial DNA gyrase and exhibits potent activity against ciprofloxacin-resistant *S. aureus in vitro* and *in vivo* using an acute pneumonia model

Luis Angel Veyna-Hurtado, Hiram Hernández-López, Denisse de Loera-Carrera, Juan Manuel Vargas-Morales, Martín Muñoz-Ortega, Lorena Troncoso-Vázquez, Alondra Bocanegra Zapata and Alberto Rafael Cervantes-Villagrana

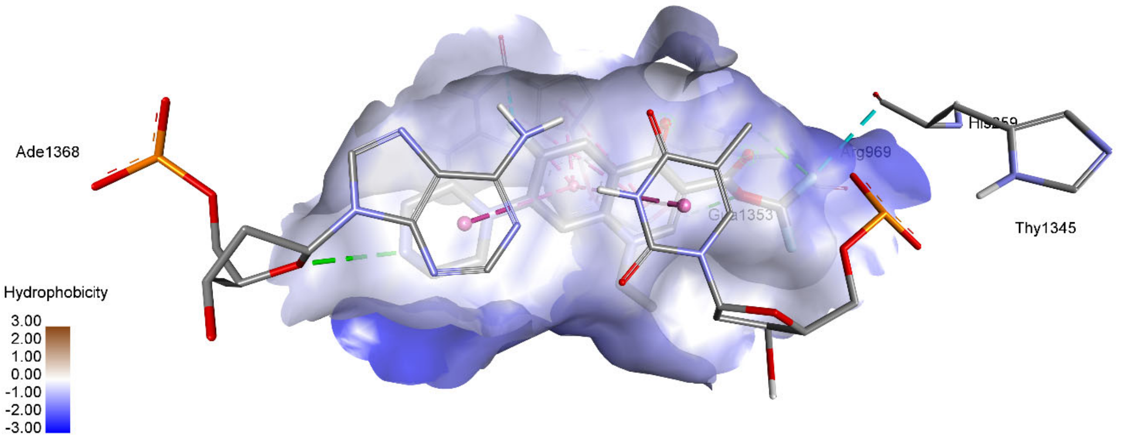

Figure S1. Hydrophobic surface of position 1 of **7a** in the interaction pocket.

Table S1. Most probable interaction positions with *S. aureus* DNA gyrase through molecular docking.

Conformation mode	7a affinity	Main interactions in pocket
1	-10.0	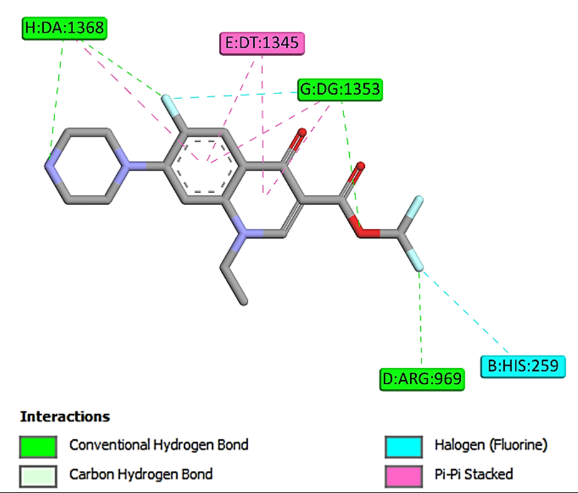
2	-9.9	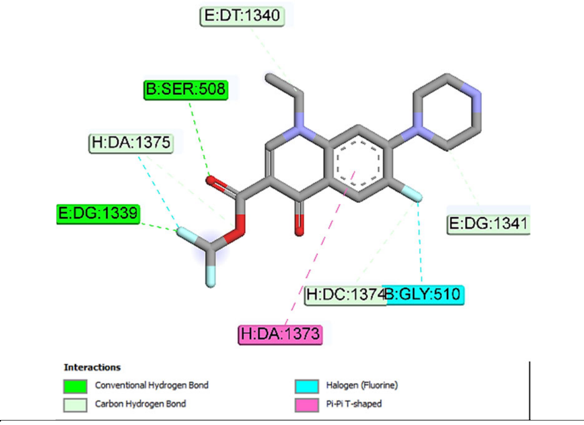
3	-9.8	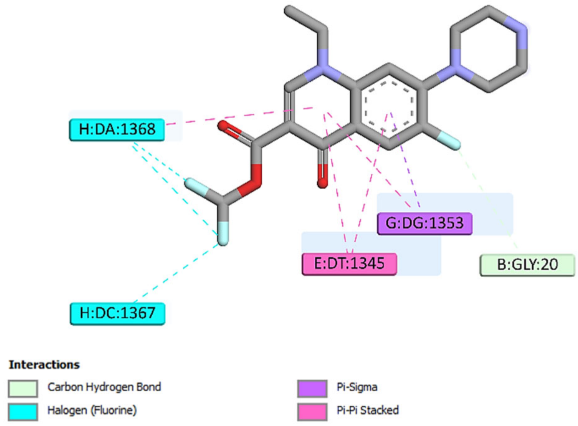

<table>
<tr><td>4</td><td>-9.5</td><td>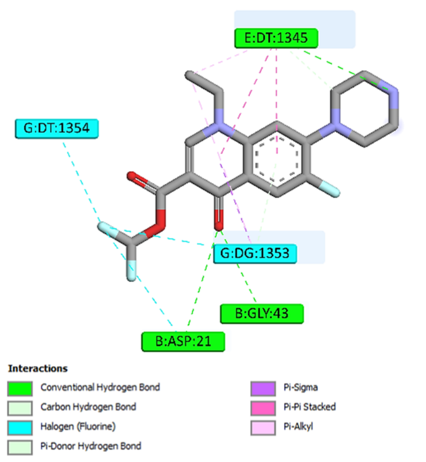
</td></tr>
<tr><td>5</td><td>-9.5</td><td>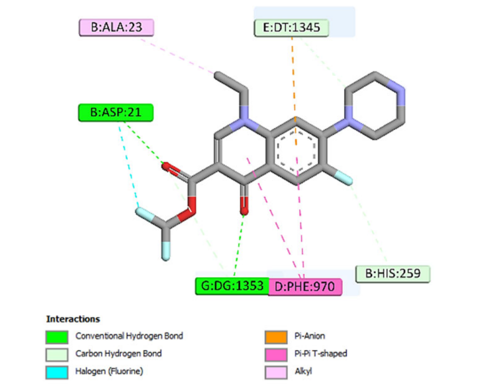
</td></tr>
<tr><td>6</td><td>-9.4</td><td>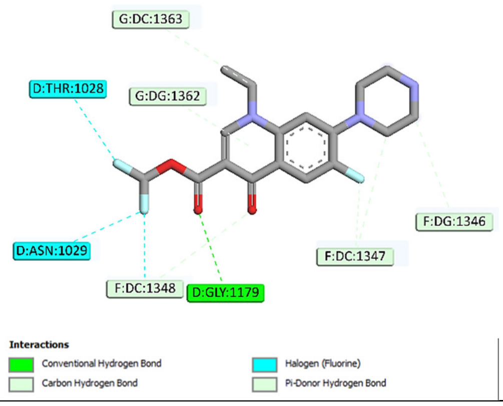
</td></tr>
</table>

7	-9.3	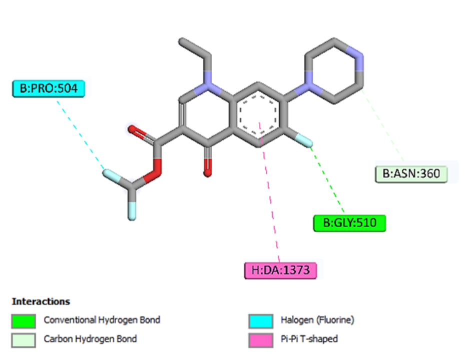B:PRO:504<br>B:ASN:360<br>B:GLY:510<br>H:DA:1373<br>Interactions<br>Conventional Hydrogen Bond<br>Carbon Hydrogen Bond<br>Halogen (Fluorine)<br>Pi-Pi T-shaped
8	-9.3	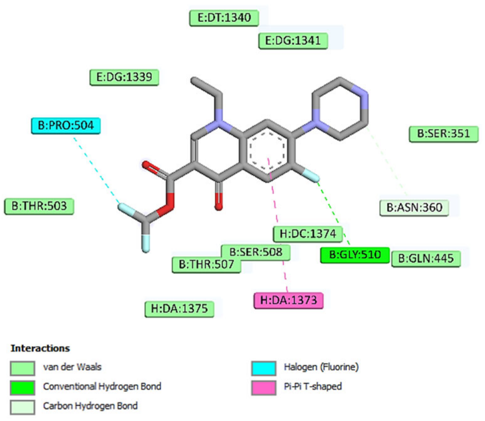E:DT:1340<br>E:DG:1341<br>E:DG:1339<br>B:PRO:504<br>B:SER:351<br>B:THR:503<br>B:ASN:360<br>H:DC:1374<br>B:SER:508<br>B:THR:507<br>B:GLY:510<br>B:GLN:445<br>H:DA:1375<br>H:DA:1373<br>Interactions<br>van der Waals<br>Conventional Hydrogen Bond<br>Carbon Hydrogen Bond<br>Halogen (Fluorine)<br>Pi-Pi T-shaped
9	-9.1	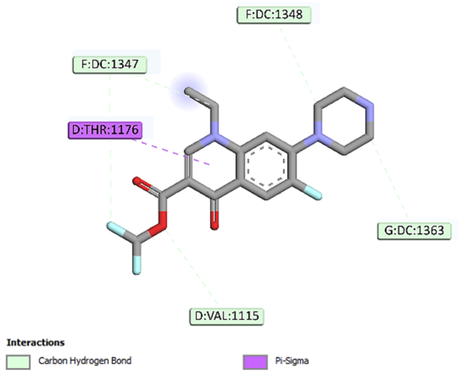F:DC:1348<br>F:DC:1347<br>D:THR:1176<br>G:DC:1363<br>D:VAL:1115<br>Interactions<br>Carbon Hydrogen Bond<br>Pi-Sigma

Table S2. Predicted metabolites of 7a through Way2drug platform

Metabolite number	Formation probability	Metabolite structure
2	0.997	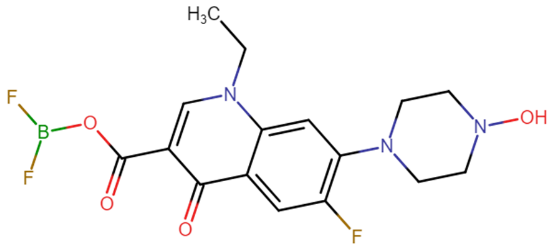
3	0.97	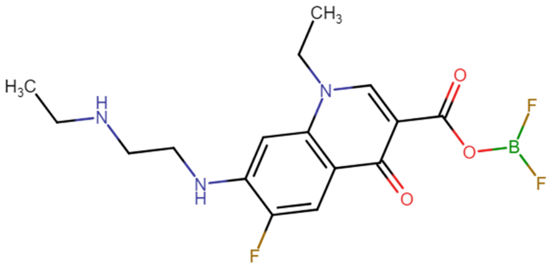
4	0.91	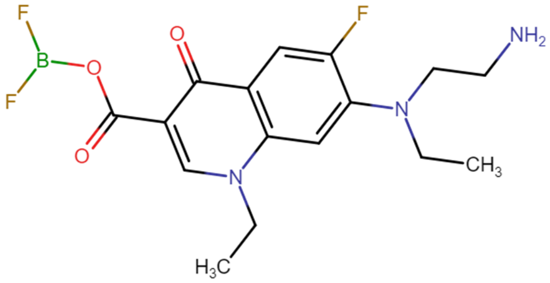
5	0.9954	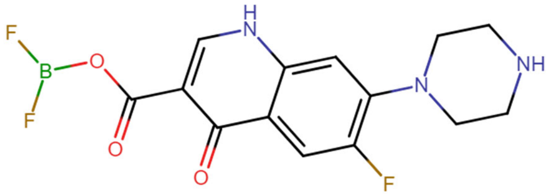

**6**	**0.99**	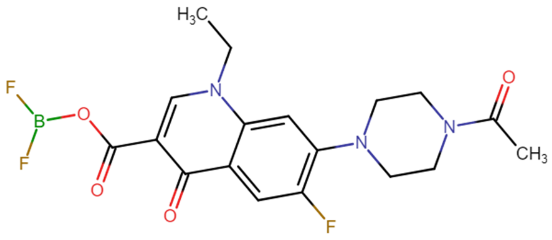
**7**	**0.76**	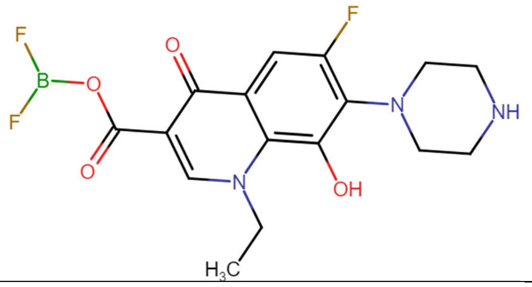
**8**	**0.62**	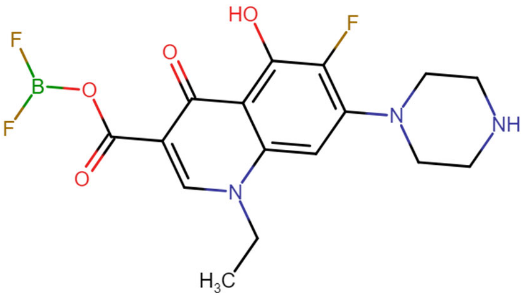
**9**	**0.59**	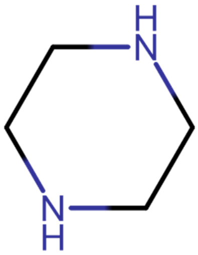
**10**	**0.59**	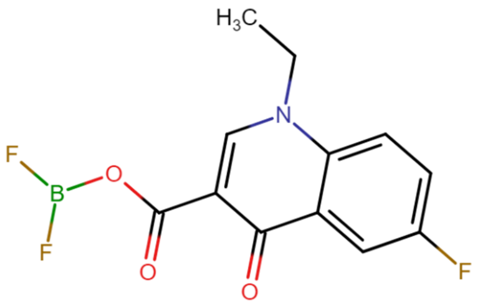

Table S3. Pharmacokinetic parameters of **7a** simulated using the SwissAMDE platform.

**Molecule**	**7a**	**Ciprofloxacin**
**Canonical SMILES**	CCn1cc2C(=O)O[B-]([O+]=c2c2c1cc(N1CCNCC1)c(c2)F)(F)F	Fc1cc2c(cc1N1CCNCC1)n(cc(c2=O)C(=O)O)C1CC1
**Formula**	C16H17BF3N3O3	C17H18FN3O3
**MW**	367.13	331.34
**#Heavy atoms**	26	24
**#Aromatic heavy atoms**	10	10
**Fraction Csp3**	0.38	0.41
**#Rotatable bonds**	2	3
**#H-bond acceptors**	7	5
**#H-bond donors**	1	2
**MR**	98.98	95.25
**TPSA**	63.57	74.57
**iLOGP**	0	2.24
**XLOGP3**	2.79	-1.08
**WLOGP**	2.15	1.18
**MLOGP**	1.28	1.28
**Silicos-IT Log P**	0.2	1.9
**Consensus Log P**	1.28	1.1
**ESOL Log S**	-4.03	-1.32
**ESOL Solubility (mg/ml)**	3.45E-02	1.57E+01
**ESOL Solubility (mol/l)**	9.41E-05	4.74E-02
**ESOL Class**	Moderately soluble	Very soluble
**Ali Log S**	-3.78	0
**Ali Solubility (mg/ml)**	6.07E-02	3.34E+02
**Ali Solubility (mol/l)**	1.65E-04	1.01E+00
**Ali Class**	Soluble	Highly soluble
**Silicos-IT LogSw**	-4.96	-3.5
**Silicos-IT Solubility (mg/ml)**	4.07E-03	1.04E-01
**Silicos-IT Solubility (mol/l)**	1.11E-05	3.13E-04
**Silicos-IT class**	Moderately soluble	Soluble

**GI absorption**	High	High
**BBB permeant**	Yes	No
**Pgp substrate**	Yes	Yes
**CYP1A2 inhibitor**	Yes	No
**CYP2C19 inhibitor**	No	No
**CYP2C9 inhibitor**	No	No
**CYP2D6 inhibitor**	Yes	No
**CYP3A4 inhibitor**	No	No
**log Kp (cm/s)**	-6.56	-9.09
**Lipinski #violations**	0	0
**Ghose #violations**	0	0
**Veber #violations**	0	0
**Egan #violations**	0	0
**Muegge #violations**	0	0
**Bioavailability Score**	0.55	0.55
**PAINS #alerts**	0	0
**Leadlikeness #violations**	1	0
**Synthetic Accessibility**	3.51	2.51
